# Liposomes Bearing Non-Bilayer Phospholipid Arrangements Induce Specific IgG Anti-Lipid Antibodies by Activating NK1.1^+^, CD4^+^ T Cells in Mice

**DOI:** 10.3390/membranes12070643

**Published:** 2022-06-23

**Authors:** Carla Landa-Saldívar, Albany Reséndiz-Mora, Sandra Sánchez-Barbosa, Anahi Sotelo-Rodríguez, Giovanna Barrera-Aveleida, Irene Nevárez-Lechuga, Iván Galarce-Sosa, Keiko Taniguchi-Ponciano, Oriana del Rocío Cruz-Guzmán, Isabel Wong-Baeza, Alejandro Escobar-Gutiérrez, Isabel Baeza, Carlos Wong-Baeza

**Affiliations:** 1 Laboratorio de Biomembranas, Departamento de Bioquímica, Escuela Nacional de Ciencias Biológicas, Instituto Politécnico Nacional, Mexico City 11340, Mexico; carla_tao@hotmail.com (C.L.-S.); cbanyrm@icloud.com (A.R.-M.); profra.sandrasb@gmail.com (S.S.-B.); anasotelor@gmail.com (A.S.-R.); aveleida@gmail.com (G.B.-A.); ire.nvz@hotmail.com (I.N.-L.); ivan.gal27@hotmail.com (I.G.-S.); 2 Laboratorio de Enzimología, Departamento de Bioquímica, Escuela Nacional de Ciencias Biológicas, Instituto Politécnico Nacional, Mexico City 11340, Mexico; 3 Unidad de Investigación Médica en Enfermedades Oncológicas, Hospital de Oncología, Centro Médico Nacional Siglo XXI (CMN-SXXI), Instituto Mexicano del Seguro Social (IMSS), Mexico City 06720, Mexico; keiko.taniguchi@hotmail.com; 4 Campus Sur, Universidad Tecnológica de México, Mexico City 09810, Mexico; orianadelrociocruzguzman@gmail.com; 5 Laboratorio de Inmunología Molecular II, Departamento de Inmunología, Escuela Nacional de Ciencias Biológicas, Instituto Politécnico Nacional, Mexico City 11340, Mexico; isa_wb@yahoo.com.mx; 6 Instituto de Diagnóstico y Referencia Epidemiológicos (InDRE), Mexico City 06600, Mexico; aescobargutierrez@yahoo.com

**Keywords:** liposomes, non-bilayer phospholipid arrangements, anti-lipid antibodies, NK1.1^+^, CD4^+^ T cells, systemic lupus erythematosus

## Abstract

Liposomes are artificial models of cellular membranes that are used as delivery systems for genes, drugs and protein antigens. We have previously used them to study the antigenic properties of their phospholipids. Here, we used them to induce the production of IgG anti-non-bilayer phospholipid arrangements (NPAs) antibodies in mice; these antibodies cause cell lysis and trigger a lupus-like disease in mice. We studied the mechanisms that lead to the production of these antibodies, and provide evidence that NK1.1^+^, CD4^+^ T cells respond to NPA-bearing liposomes and deliver the help required for specific B cell activation and antibody class-switching to IgG. We found increased numbers of IL-4-producing NK1.1^+^, CD4^+^ T cells in the secondary lymphoid organs of mice administered with NPAs, and these cells also expressed CD40L, which is required for B cell activation. Additionally, we isolated and purified NK1.1^+^, CD4^+^ T cells from spleens and determined that they over-expressed 40 genes, which are key players in inflammatory processes and B cell stimulation and have TRAF6 and UNC39B1 as key nodes in their network. These results show that liposomes are membrane models that can be used to analyze the immunogenicity of lipids.

## 1. Introduction

Liposomes are vesicular structures mainly made of phospholipids. Structurally, they resemble the lipid bilayer membrane of living cells and they are widely used as efficient delivery systems for genes [1], drugs [2,3] or protein antigens [4]. Moreover, liposomes are used to enhance the immune response; in some cases, they deliver bioactive lipids to enhance phagosome maturation, which is a critical step in the effector function of innate immunity [5]. In other cases, liposomes are vaccine adjuvants that induce the production of antibodies [6]. For example, in the mRNA-based vaccines against COVID-19, the mRNA that encodes the coronavirus spike protein is encapsulated in liposomes [7,8]. These liposomes induce a transient local inflammation, which triggers the recruitment and activation of immune cells, including antigen-presenting cells that process the protein and present it to helper T cells. Activated T cells cooperate and stimulate B cells to produce anti-spike protein antibodies [9]. In our research group, we have used liposomes to induce the production of IgG anti-non-bilayer phospholipid arrangements (NPAs) antibodies in mice [10,11]. These antibodies are found in some patients with Systemic Lupus Erythematosus (SLE), and trigger a disease that resembles human lupus (lupus-like disease) in BALB/c and NIH mice strains [10].

SLE is a chronic and multisystemic autoimmune disease with a complex etiology. SLE is characterized by the presence of autoantibodies, immune complexes and inflammation, which leads to tissue damage in the cartilages, skin, kidneys, heart, lungs, blood vessels and brain [12,13]. SLE is a result of multiple alterations in the innate and adaptive immune systems, comprising disorders of immune tolerance, hyperactivation of T and B cells and decreased clearance of immune complexes and apoptotic cells. B cell hyperactivity leads to the excessive production of multiple autoantibodies, which represents one of the immunological hallmarks of SLE [13,14,15]. Several animal models have been used to study the pathogenesis of this disease [16]. The model of lupus induced by NPAs in BALB/c and NIH mice has clinical and pathological characteristics that closely resemble those of SLE patients, including anti-nuclear, anti-histone and anti-coagulant antibodies. These mice also develop anti-cardiolipin and anti-NPA antibodies, weight loss, glomerulonephritis and facial lesions (similar to the typical “butterfly” rash in SLE patients) [10,17,18]. This lupus model is produced by the administration of NPA-bearing liposomes induced and stabilized with drugs, such as chlorpromazine, promazine, procainamide or hydralazine [10,17]; these drugs also trigger a lupus-like disease in humans. NPAs are three-dimensional structures that can emerge as protuberances on the surface of cells or liposomes, when anionic phospholipids with a conical shape, such as phosphatidate, interact with these drugs, which have positive charge. So, the interaction between anionic conical lipids and these drugs generates stable NPAs [17]. Stabilized NPAs induce the production of IgG anti-NPA antibodies that are mainly generated via germinal centers in secondary lymphoid organs [11].

The formation of germinal centers is a key step in the production of IgG anti-protein antibodies. Germinal centers are usually organized as a result of direct interactions of a protein antigen with particular subsets of T-helper (CD4^+^) and B cells. These interactions depend on the binding of CD40L on CD4^+^ T cells with CD40 on B cells, and on the local cytokine environment created by activated CD4^+^ T cells [13,19]. CD4^+^ T cells are specific for peptides presented by major histocompatibility complex (MHC) class II molecules on antigen-presenting cells. However, classical MHC molecules do not present lipid antigens. NKT cells are lymphocytes restricted to the recognition of lipid antigens presented by CD1d molecules, an antigen-presenting molecule similar to MHC class I molecules, on B cells or dendritic cells [20], with both innate and adaptive functions [20,21]. Human and murine NKT cells are a distinct subset of T cells that express both a T cell receptor (TCR α/β and CD3) and NK cell lineage markers [NK1.1 (CD161), CD122 (IL-2Rβ) and various Ly49 molecules] [22,23]. NK1.1 expression on CD4^+^ T cells can be used for the identification of NKT cells [24], and these cells represent 1–2% of the spleen lymphocytes in mice [25,26,27]. NKT cells respond rapidly to antigenic stimulation by secreting IL-4 and IFN-γ, which results in the activation of numerous immune cells, including dendritic cells, NK cells, B cells and CD4^+^ and CD8^+^ T cells [24]. NKT cells interact through their TCR with CD1d-expressing B cells, and this interaction leads to B cell proliferation and antibody production, and to increased NKT cell activation [20,21,25]. This cooperation requires the expression of CD40 on B cells and of CD40L on NKT cells; likewise, it needs the production of IL-4 and IFN-γ by activated NKT cells, two essential cytokines that induce the class-switching of antibodies against lipid antigens [20].

Since IgG anti-NPA antibodies are mainly produced via germinal center, this implies that the NPA-specific germinal center B cells interact with a T-lineage cell. So, in this work we analyzed the proportion, cytokine production and gene expression profile of NK1.1^+^, CD4^+^ T cells, in order to determine if these cells provide the help required by germinal center B cells to produce IgG anti-NPA antibodies. We used NPA-bearing liposomes to induce the production of IgG anti-NPA antibodies in C57BL/6 mice, and we analyzed NK1.1^+^, CD4^+^ T cells from the spleen and mesenteric lymph nodes of these mice to evaluate their activation and their possible involvement in helping germinal center B cells to proliferate and to produce IgG anti-NPA antibodies. We also evaluated the expression of genes related to the innate and adaptive immune responses in NK1.1^+^, CD4^+^ T cells from the spleens of these mice, and analyzed the protein–protein interaction network of the most up-regulated genes. This study demonstrates the use of liposomes as membrane models to analyze the immunogenicity of lipids. Our results contribute to the understanding of the mechanisms that lead to the production of IgG anti-NPA antibodies, which can trigger the development of a disease in mice that is similar to human SLE.

## 2. Materials and Methods

### 2.1. Ethics

The animal studies were carried out in the National School of Biological Science of the National Polytechnic Institute in accordance with the principles of the “Guide for the Care and Use of Laboratory Animals” from the US National Institutes of Health [28]. The protocol was approved by the Bioethics Committee of our institution.

### 2.2. Preparation and Characterization of Liposomal Antigens

Liposomes were formed as previously described by a modified reverse-phase evaporation method [17,18], with egg yolk L-α-phosphatidylcholine (PC), a lipid with neutral charge and cylindrical shape (CAS No: 8002-43-5, molecular weight: 311.22 g/mol) (Sigma Aldrich, St. Louis, MO, USA), and egg yolk L-α-phosphatidic acid (PA), a lipid with negative charge and conical shape (MDL No: MFCD00063023, molecular weight: 194.06 g/mol) (Sigma Aldrich), in a 2:1 molar ratio. Phospholipids (nine micromoles of the mixture) were dissolved in 1 mL of diethyl ether, 330 μL of TS (10 mM Tris-HCl, 1 mM NaCl, pH 7) were added and the mixture was sonicated three times (5 s sonication followed by 30 s resting period) in a Lab Supply G112SPI sonicator (Laboratory Supplies, Hicksville, NY, USA). Subsequently, diethyl ether was totally removed under a stream of oxygen-free dry nitrogen at reduced pressure, using an evaporator at 37 °C. Then, TS buffer was added to obtain a final volume of 1 mL, which was filtered with a 0.45 µm Millipore membrane (Billerica, MA, USA), obtaining a suspension of smooth liposomes.

To induce NPAs, smooth liposomes were incubated at 37 °C for 30 min with promazine (CAS No: 53-60-1, molecular weight: 320.88 g/mol) (Sigma Aldrich) as inducer, obtaining a suspension of NPA-bearing liposomes [17,18]. To determine the optimal promazine concentration to induce NPA on liposomes, promazine concentrations of 6, 8 and 10 mM were tested. The formation of NPAs in liposomes was demonstrated by flow cytometry in a LSR Fortessa cytometer (Becton Dickinson, San Jose, CA, USA) with FACSDiva software, with a compensation threshold in FSC-H of 52 V, FSC set at E00, SSC at 401 V and FL1 at 748 V [10]. 10,000 events were acquired for each sample. Results were reported as numbers of liposomes, and as SSC histograms and FSC and SSC dot plots in logarithmic scales [10]. Liposome size was determined by nanoparticle tracking analysis in a Nanosight NS3000 (Malvern Panalytical, Malvern, UK) with NTA 3.2.16 software. Mean sizes were reported in nm.

In previous studies, we have demonstrated that smooth and NPA-bearing liposomes do not generate cytotoxicity on HEK293 cells or bone marrow-derived macrophages [18], and that smooth liposomes do not cause tissue damage to mice after up to 6 months of monthly administration [10,17].

### 2.3. Induction of IgG Anti-NPA Antibodies in C57BL/6 Mice

To induce the production of IgG anti-NPA antibodies, we used six-week-old female C57BL/6 mice. This method has been previously described in BALB/c mice [10,17]. Each mouse received 100 µL of smooth liposomes, or 100 µL of NPA-bearing liposomes (induced with 8 mM promazine), by intrasplenic injection on days 1 and 16. One day before the first intrasplenic injection, mice received 100 µL of complete Freund’s adjuvant (Sigma Aldrich) diluted 1:2 in TS buffer, and before the second intrasplenic injection, mice received 100 µL of incomplete Freund’s adjuvant (Sigma Aldrich), diluted 1:2 in TS buffer, both by intraperitoneal injection. On days 8 and 24 after the first intrasplenic injection, mice were injected intraperitoneally with 100 µL of smooth liposomes or of NPA-bearing liposomes, respectively [11,18]. Some mice were then injected intraperitoneally with 100 µL of smooth liposomes or of NPA-bearing liposomes each week for 4 months, and were then euthanized. Other mice were euthanized on days 10, 20 or 30 after the first intrasplenic injection. Spleens and mesenteric lymph nodes were obtained from the euthanized mice, and were disaggregated to obtain cell suspensions in FACS (fluorescence-activated cell sorting) buffer, consisting of PBS (phosphate buffered saline) pH 7.2 (Gibco, Gran Island, NY, USA) with 1% bovine serum albumin (Biowest, Nuaillè, France) and 0.01% sodium azide (Sigma Aldrich), for subsequent flow cytometry assays.

### 2.4. Detection of IgG Anti-NPA, Anti-Cardiolipin and Anti-Histone Antibodies in Mice Sera

Mice were bled from the facial vein before, and every 15 days for four months after, the first intrasplenic injection of smooth liposomes or of NPA-bearing liposomes. Sera were incubated at 56 °C for 30 min to inactivate complement proteins and then aliquoted at −70 °C until use. IgG anti-NPA, anti-cardiolipin and anti-histones antibodies were determined in these sera by ELISA, as previously described [10,11]. Briefly, to detect anti-NPA antibodies, microtiter plates with 96 flat-bottom wells (Costar Co., Cambridge, MA, USA) were coated with smooth liposomes or with liposomes bearing promazine-induced NPAs (0.1 μmol in 100 μL TS buffer) at room temperature for 12 h. After incubation, plates were blocked with 200 μL per well of 8% fetal bovine serum (Gibco) in TS buffer for 1 h at room temperature and washed, and 100 μL of inactivated mice sera were added. H308 antibody (an IgM anti-NPA monoclonal antibody) was added as a positive control for NPA detection. After incubation for 1 h at 37 °C, the plates were washed 5 times and 100 μL of goat anti-mouse IgG peroxidase-conjugated antibody (Sigma Aldrich) at a dilution of 1:2000 were added to the assay wells, and 100 μL of goat anti-mouse IgM peroxidase-conjugated antibody (Sigma Aldrich) were added to the positive control wells. The plates were incubated for 1 h at 37 °C and washed 3 times before adding 100 μL per well of peroxidase substrate. After 20 min at 37 °C, the reaction was stopped by addition of sulfuric acid 2.5 M and absorbances were read at 492 nm in a Labsystem Multiskan MS reader (MTX Labsystems, Vienna, VI, USA). Each sample was assayed in triplicate. Anti-cardiolipin and anti-histone antibodies were measured by ELISA, as described before [10,29].

### 2.5. Analysis of Spleen and Mesenteric Lymph Node NK1.1^+^, CD4^+^ T Cells

Cell suspensions were prepared from the spleens and mesenteric lymph nodes of C57BL/6 mice that received smooth liposomes or NPA-bearing liposomes, as described above, and were filtered through a 70 µm cell strainer (Becton Dickinson). Erythrocytes in the spleen samples were eliminated by hypotonic lysis, followed by re-suspension in FACS buffer. Cell suspensions were blocked with a universal blocker (Block Biogenex, San Ramon, CA, USA) diluted 1:10 in PBS, at 4 °C for 10 min, and were subsequently washed with PBS [11]. The cell suspensions of spleens and mesenteric lymph nodes were quantified with the Neubauer chamber method with trypan blue as exclusion dye. Two million live cells were incubated with fluorochrome-coupled antibodies to identify the surface phenotype characteristic of NK1.1^+^, CD4^+^ T cells. Cells were stained with anti-CD3/FITC (clone:17A2, isotype: rat IgG2b, κ/FITC), anti-NK1.1/APC (clone:PK136, isotype: mouse IgG2a, κ/APC), anti-CD4/Pacific Blue (clone: GK1.5, isotype: rat IgG2b, κ/Pacific Blue) and anti-CD40L/PE (clone:MR1, isotype: 5rmenian hamster IgG/PE) antibodies (BioLegend, San Diego, CA, USA). The anti-CD3, anti-NK1.1 and anti-CD4 antibodies and their isotypes controls were used at a final concentration of 10 mg/mL, while the anti-CD40L antibody and its isotype were used at a final concentration of 2.5 mg/mL. After 30 min at 4 °C, the cells were washed and fixed with 1% paraformaldehyde (Sigma Aldrich). Stained NK1.1^+^, CD4^+^ T cells were analyzed in a LSR Fortessa flow cytometer (Becton Dickinson). 400,000 events were acquired from the gate that corresponds to lymphocytes according to their size (FSC-A) and granularity (SSC-A). Data were analyzed with FlowJo 10.8.1 software (Tree Star, Inc., Ashland, OR, USA). Results were reported as absolute cell numbers, obtained from the cell count with the Neubauer chamber method and the flow cytometry analysis. Unstained cells, compensation controls and fluorescence-minus-one controls were used as appropriate.

To evaluate cytokine production in NK1.1^+^, CD4^+^ T cells, spleen or mesenteric lymph node cells were treated with 0.7 µL/mL monensin (Becton Dickinson) for 5 h at 37 °C. After this incubation, the cells were washed and fluorochrome-coupled antibodies (anti-CD3/FITC, anti-CD4/Pacific Blue and anti-NK1.1/APC) were added. The cells were then incubated for 30 min at 4 °C. Subsequently, 100 µL of Cytofix/Cytoperm (Becton Dickinson) was added, and the cells were incubated for 10 min at 4 °C. Cells were washed with Perm/Wash 1X buffer (Becton Dickinson) and anti-IFN-γ/PE (clone: XMG1.2, isotype: rat IgG1, κ/PE), anti-IL-4/PerCP-Cy5.5 (clone:11B11, isotype: rat IgG1, κ/PerCP-Cy5.5) and anti-IL17/APC-Cy7 (clone:TC11-18H10.1, isotype: rat IgG1, κ/APC-Cy7) antibodies (BioLegend) were added. The anti-IFN-g and anti-IL-4 antibodies and their isotypes controls were used at a final concentration of 2.5 mg/mL, while the anti-IL-17 antibody and its isotype were used at a final concentration of 5 mg/mL. After 30 min at 4 °C, the cells were washed and fixed with 1% paraformaldehyde. The stained cells were acquired in a LSR Fortessa flow cytometer, and data were analyzed with FlowJo 10.8.1 software. Unstained cells, compensation controls and fluorescence-minus-one controls were used as appropriate.

### 2.6. In Vitro Re-Stimulation of NK1.1^+^, CD4^+^ T Cells

Six-week-old female C57BL/6 mice received smooth liposomes or liposomes bearing NPAs (induced with 8 mM promazine) as described above, and were euthanized 20 days after the first intrasplenic injection of the liposomal antigen. The spleens and mesenteric lymph nodes were extracted from the euthanized mice to obtain cell suspensions in FACS buffer. Two million cells were placed in each well of a 24-well plate with 1 mL of RPMI 1640 supplemented with 5% complement-free fetal bovine serum (Gibco), 2 mM L-glutamine and 0.1% penicillin–streptomycin (Gibco, 100 U/mL-100 mg/mL). Subsequently, smooth liposomes or NPA-bearing liposomes were added at a final concentration of 0.05 μmol, and the cells were incubated at 37 °C and 5% CO_2_ for 30, 90 or 120 min. The cells were analyzed by flow cytometry as described above, with staining for CD3, CD4, NK1.1/APC, IFN-γ, IL-4 and IL-17.

### 2.7. Gene Expression Analysis of NK1.1^+^, CD4^+^ T Cells

Spleens were obtained 20 days after the first administration of the liposomal antigens to mice. Extracted spleens were disaggregated and cells were re-suspended and passed through a 70 µm cell strainer. Ten spleens were pooled for each liposomal antigen condition (smooth liposomes or NPA-bearing liposomes). Subsequently, erythrocytes were eliminated by hypotonic lysis and the remaining cells were re-suspended in FACS buffer. The number and viability of cells was obtained with the Neubauer chamber method. To enrich CD4^+^ T cells, 2 × 10^9^ spleen cells were placed in MACS buffer [PBS pH 7.2, 0.5% bovine serum albumin and 2 mM EDTA] and anti-CD4 microbeads [MicroBeads Kit (Miltenyi Biotec, Auburn, CA, USA)] were added. The cell suspension was then passed through a MACS LS column (Miltenyi Biotec) in the VarioMACS separator (Miltenyi Biotec). After the CD4-negative cells were eluted, the CD4-positive cells were released from the column, and they were washed and re-suspended in FACS buffer. The number of viable CD4^+^ cells was determined with the Neubauer chamber method.

The enriched CD4^+^ T cells were stained with anti-CD3/FITC and anti-NK1.1/APC antibodies, and NK1.1^+^, CD4^+^ T cells were then purified from the CD4^+^-positive cells by flow cytometry (FACSAria III flow cytometer, Becton Dickinson). The purified NK1.1^+^, CD4^+^ T cells were washed with PBS. Total RNA was extracted from each sample of NK1.1^+^, CD4^+^ T cells with the TRIzol method (Invitrogen, Carlsbad, CA, USA). RNA concentration was determined in a NANODROP 2000c (Thermo Fisher Scientific, Waltham, MA, USA). The integrity of the RNA samples was confirmed by agarose gel electrophoresis. cDNA synthesis was performed from 115 ng of RNA, using the RT^2^ First Strand kit (Qiagen, Germantown, MD, USA). Each cDNA was amplified by real-time PCR, using the RT^2^ SYBR-Green Rox qPCR master mix (Qiagen) and an RT^2^ Profiler PCR array (Qiagen) that analyzes 84 genes related to the innate and adaptive immune responses. Amplification was carried out in a 7500 Fast thermal cycler (Applied Biosystems, Waltham, MA, USA). The experiments were performed in triplicates. The C_T_ values were exported to an Excel sheet and analyzed in the PCR array data analysis web portal (available at www.SABioscience.com/pcrarraydataanalysis.php accessed on 18 October 2021), where the gene expression quantification was automatically performed with the ΔΔC_T_ method. The selected housekeeping gene was β-actin, since its expression was not modified between the analyzed conditions.

### 2.8. Protein–Protein Interaction (PPI) Network Analysis in NK1.1^+^, CD4^+^ T Cells

The 10 most up-regulated genes in NK1.1^+^, CD4^+^ T cells from mice that received NPA-bearing liposomes, compared with mice that received smooth liposomes, were mapped with the Search Tool for the Retrieval of Interacting Genes/Proteins (STRING 10.5) (http://string-db.org accessed on 8 November 2021), an online database comprising known and predicted interactions among proteins [30]; only the interactions with a combined score >0.7 (high confidence) were considered as significant. PPI pairs were visualized using Cytoscape software 3.4.0 (http://www.cytoscape.org/ accessed on 22 November 2021). To obtain the significant nodes/proteins [31], the highly connected proteins were identified in Cytoscape by calculating the degree of connectivity (number of line connections between nodes) and the betweenness value (fraction of the number of shortest paths that pass through each node, this parameter measures how often nodes occur on the shortest paths between other nodes) of each node with a degree cutoff of ≥2. The main connected components of the PPI network were laid out by degree values.

### 2.9. Statistical Analysis

Statistical analysis was performed using GraphPad Prism version 9 (GraphPad, San Diego, CA, USA). For ELISA results, the comparison of the autoantibody titers of mice administered with smooth liposomes or with NPA-bearing liposomes was performed with Mann–Whitney U test, with significance set at *p* < 0.05, and the results are presented as mean and standard deviation. For flow cytometry results, cell number comparison was performed with Kruskal–Wallis test with Dunn’s post-test, with significance set at *p* < 0.05, and the results are presented as individual data with median. For PCR array results, relative quantification was performed with the ΔΔC_T_ method. The *p* values were calculated with *t*-tests of the 2^(−ΔΔCT)^ values for each gene.

## 3. Results

### 3.1. Liposomes Bearing Promazine-Induced NPAs Induce the Production of IgG Anti-NPA Antibodies in C57BL/6 Mice

**Figure 1:**
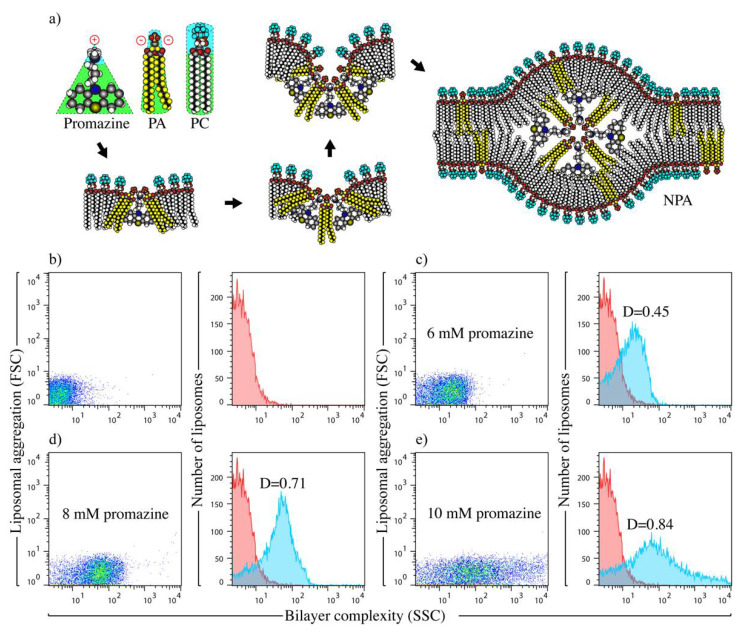
Induction and formation of one NPA, and characterization of liposomal antigens. (**a**) Schematization of how promazine induces a lipid re-arrangement that forms an inverted micelle, which is the center of the NPA. Promazine is an amphipathic molecule with a triangular shape and a positive charge, that can interact with the amphipathic lipids L-α-phosphatidate (PA) and L-α-phosphatidylcoline (PC). However, it preferably interacts with phosphatidate, which has a conical shape and two negative charges, and not with phosphatidylcoline, which has a cylindrical shape and no charge. (**b**–**e**) Flow cytometric characterization of liposomes. Phosphatidylcholine/phosphatidate liposomes (2:1 molar ratio) were incubated at 37 °C for 30 min with TS buffer or with the NPA-inducer promazine. Changes in bilayer complexity (SSC) are shown as dot plots and histograms. The dot plot in (**b**) and red color histograms in (**b**–**e**) represent smooth liposomes. Dot plots in (**c**–**e**) and blue histograms in (**c**–**e**) are liposomes with promazine-induced NPAs at a promazine concentration of (**c**) 6, (**d**) 8 and (**e**) 10 mM. A D > 0.5 value from the Kolmogorov–Smirnov test indicates a statistically significant difference between liposome samples, and reveals the presence of NPA in liposomes. One experiment representative of three is shown.

The drug promazine is an amphipathic molecule with a triangular shape and a positive charge, which preferably interacts with PA (conical lipid with two negative charges) instead of PC (cylindrical lipid with no charges) in liposomes made of PC/PA. This interaction induces a lipid re-arrangement that forms an inverted micelle that is the center of the NPA (Figure 1a). NPAs cause an increase in bilayer complexity, which can be detected by flow cytometry in the side scatter detector [10,17,18]. PC/PA liposomes in TS buffer showed the characteristic low-complexity profile for smooth liposomes (Figure 1b). After incubation with promazine, liposomes showed an increase in bilayer complexity (Figure 1c–e) as the drug concentration increases, compared with smooth liposomes.

When the bilayer complexity of smooth liposomes and of NPA-bearing liposomes were compared with the Kolmogorov–Smirnov test, the liposomes with NPAs induced by 6 mM promazine (Figure 1c) had a D value less than 0.5 (D = 0.45), which indicates the absence of NPAs in these liposomes. However, the liposomes with NPAs induced by 8 and 10 mM promazine (Figure 1d,e) had D values higher than 0.5 (D = 0.71 and D = 0.84, respectively), which indicates the presence of NPAs in those liposomes. However, at concentrations of 10 mM, the liposome population was highly heterogeneous (Figure 1e), so 8 mM promazine was selected, because it induces a more homogeneous population of NPA-bearing liposomes (Figure 1d). We have used the same criteria to select the concentration of other NPA-inducing drugs, such as chlorpromazine, procainamide and hydralazine [10,17]. The smooth liposomes present a mean size of 256.7 ± 4.3 nm, while the NPA-bearing liposomes induced by 6, 8 and 10 mM promazine showed a mean size of 249.8 ± 3.8, 250.7 ± 3.5 and 253.5 ± 4.0, respectively.

**Figure 2:**
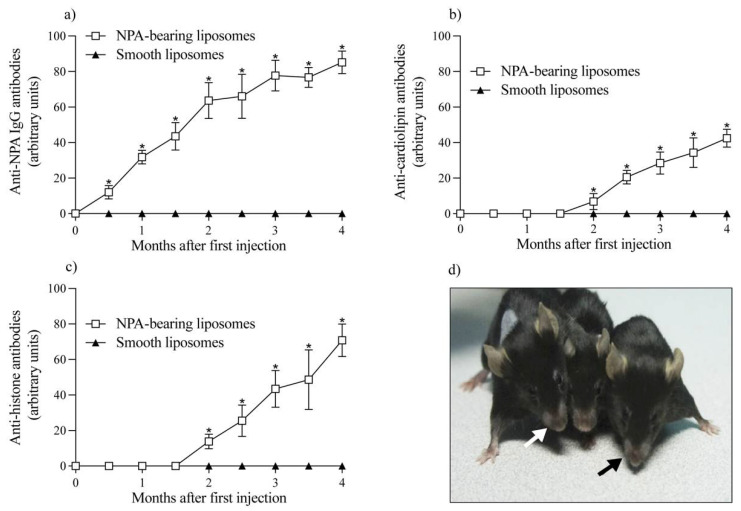
C57BL/6 mice injected with NPA-bearing liposomes produce autoantibodies and develop facial lesions, alopecia and deformation of limbs. (**a**) IgG anti-NPA, (**b**) anti-cardiolipin and (**c**) anti-histone antibodies were determined by ELISA in the sera of mice injected with smooth liposomes or with NPA-bearing liposomes, at the indicated times. One experiment representative of three is shown. Asterisks represent statistically significant differences between autoantibodies from mice injected with smooth vs. NPA-bearing liposomes (* *p* < 0.05). Each symbol represents a 6-mice sample. Female mice that received NPA-bearing liposomes showed alopecia, deformation of limbs and (**d**) facial lesions (black and white arrows) on day 20 after the first administration of NPAs.

We have previously used NPA-bearing liposomes to induce a lupus-like disease in BALB/c mice, with the production of IgG anti-NPA antibodies [11]. IgG anti-NPA antibodies were detected in the serum of C57BL/6 mice after the administration of liposomes bearing promazine-induced NPAs, but not after the administration of smooth liposomes (Figure 2a). Anti-NPA antibodies were detected since day 15 after the first administration of NPA-bearing liposomes, and they progressively increased until the fourth month, showing a significant statistical difference compared with mice that received smooth liposomes. The presence of anti-NPA antibodies triggers a disease resembling human lupus in BALB/c mice [10,17]. This disease is also started in C57BL/6 mice, because anti-cardiolipin (Figure 2b) and anti-histone (Figure 2c) antibodies were detected 1.5 months after the anti-NPA antibodies were first detected in mice that received NPA-bearing liposomes, but not smooth liposomes. The titers of both antibodies increased over time in mice that received NPA-bearing liposomes and show a significant statistical difference with mice that received smooth liposomes. These results suggest that anti-NPA antibodies caused cell lysis and exposed intracellular antigens to the immune system, which generated anti-cardiolipin and anti-histone auto-antibodies. The auto-antibody titers of C57BL/6 mice were comparable to the titers previously reported in BALB/c mice. In addition, these C57BL/6 female mice showed alopecia, deformation of limbs and facial lesions (Figure 2d) on day 20 after the first administration of NPA-bearing liposomes, which are some of the characteristics of the lupus-like disease described in BALB/c mice.

### 3.2. NK1.1^+^, CD4^+^ T Cells Are Activated in Mice That Produce Anti-NPA Antibodies, and These Cells Produce IL-4 in Response to NPAs

**Figure 3:**
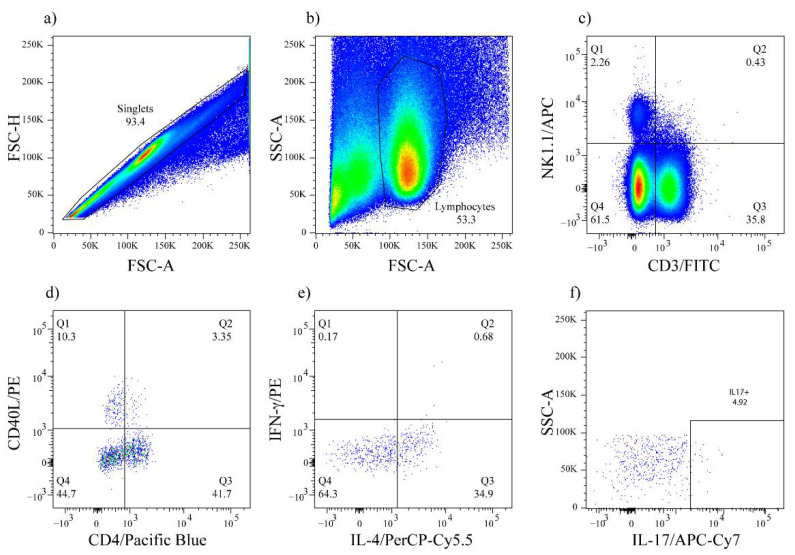
Flow cytometry strategy for the identification and characterization of NK1.1^+^, CD4^+^ T cells. (**a**) Individual events were selected, and the (**b**) lymphocyte gate was chosen by size (FSC-A) and granularity (SSC-A). Subsequently, (**c**) positive cells for CD3 and NK1.1 were selected, and two different phenotypes were evaluated, (**d**) CD3^+^, NK1.1^+^, CD4^+^, CD40L^−^ (NK1.1^+^, CD4^+^ T cells) and CD3^+^, NK1.1^+^, CD4^+^, CD40L^+^ (activated NK1.1^+^, CD4^+^ T cells). The production of (**e**) IL-4, IFN-γ and (**f**) IL-17 was subsequently evaluated on NK1.1^+^, CD4^+^ T cells.

In order to produce IgG antibodies, B cells require the cooperation of a T cell subset that expresses CD40L and produces class-switching cytokines, such as IL-4 or IFN-γ. NKT cells are lymphocytes restricted to the recognition of lipid antigens presented by CD1d molecules [20], and could provide the cooperation required to produce the IgG anti-NPA antibodies detected in C57BL/6 mice that received NPA-bearing liposomes. Here we evaluated the number of NK1.1^+^, CD4^+^ T cells and their activation in C57BL/6 mice that produce anti-NPA antibodies. Spleen and mesenteric lymph nodes cell suspensions were obtained from mice administered with smooth liposomes or with NPA-bearing liposomes, and were stained with anti-CD3, anti-NK1.1, anti-CD4 and anti-CD40L fluorochrome-coupled antibodies to identify NK1.1^+^, CD4^+^ T cells and activated NK1.1^+^, CD4^+^, CD40L^+^ T cells by flow cytometry (Figure 3). The flow cytometry strategy used was as follows: after selecting individual events (Figure 3a), the lymphocyte gate was selected according to their size and granularity (Figure 3b), and positive cells for CD3 and NK1.1 were selected (Figure 3c). Two different phenotypes were evaluated: CD3^+^, NK1.1^+^, CD4^+^, CD40L^−^ (NK1.1^+^, CD4^+^) and CD3^+^, NK1.1^+^, CD4^+^, CD40L^+^ (activated NK1.1^+^, CD4^+^) T cells (Figure 3d). A similar procedure was followed to analyze IFN-γ, IL-4 (Figure 3e) and IL-17 (Figure 3f) production by NK1.1^+^, CD4^+^ T cells.

**Figure 4:**
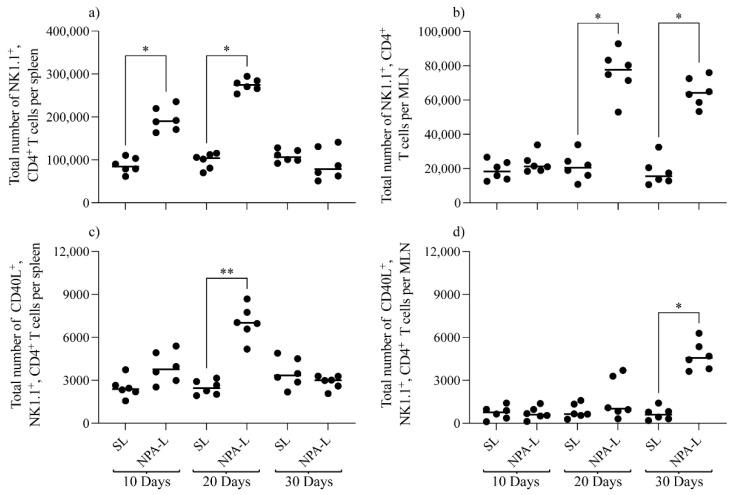
NK1.1^+^, CD4^+^ T cells and activated NK1.1^+^, CD4^+^, CD40L^+^ T cells numbers increase in the spleen and mesenteric lymph nodes of mice that produce IgG anti-NPA antibodies. (**a**,**b**) Number of NK1.1^+^, CD4^+^ T cells and (**c**,**d**) activated NK1.1^+^, CD4^+^, CD40L^+^ T cells from (**a**,**c**) spleens and (**b**,**d**) mesenteric lymph nodes (MLN), at 10, 20 and 30 days after the injection of smooth liposomes or NPA-bearing liposomes to mice. One experiment representative of three is shown. * *p* < 0.05; ** *p* < 0.01; SL, smooth liposomes; NPA-L, liposomes bearing promazine-induced NPAs.

The flow cytometry analysis revealed that the number of NK1.1^+^, CD4^+^ T cells from both the spleen and mesenteric lymph nodes were significantly increased in mice that produce anti-NPA antibodies, compared with mice administrated with smooth liposomes, but with different kinetics, as the statistically significant difference (*p* < 0.05) was observed on days 10 and 20 for the spleen (Figure 4a) and on days 20 and 30 for mesenteric lymph nodes (Figure 4b). In these mice, activated NK1.1^+^, CD4^+^, CD40L^+^ T cells significantly increased (*p* < 0.001) in the spleen on day 20 (Figure 4c) and in the mesenteric lymph nodes on day 30 (*p* < 0.05) (Figure 4d).

**Figure 5:**
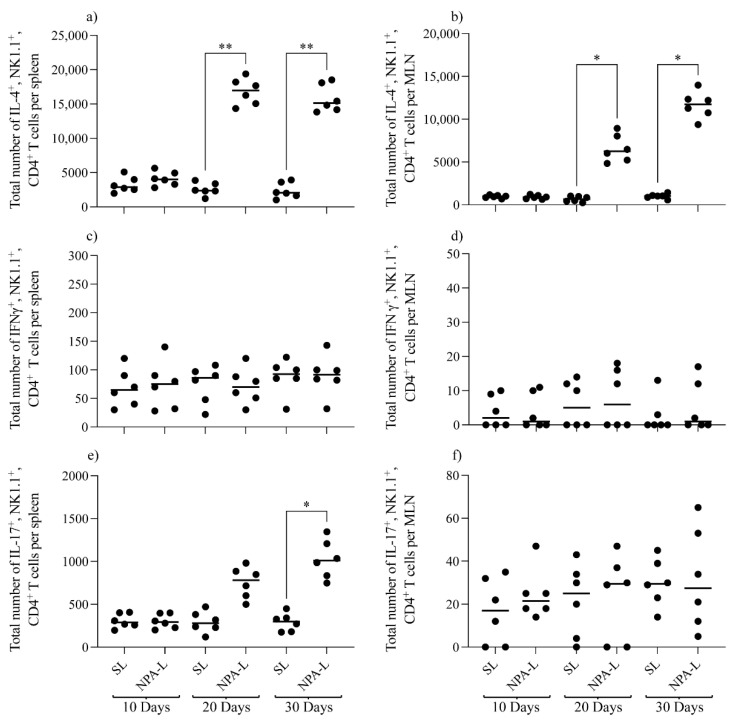
IL-4 production by NK1.1^+^, CD4^+^ T cells from the secondary lymphoid organs of mice that produce IgG anti-NPA antibodies. Numbers of (**a**,**b**) IL-4- (**c**,**d**) IFN-γ− or (**e**,**f**) IL-17-producing NK1.1^+^, CD4^+^ T cells from the (**a**,**c**,**e**) spleens and the (**b**,**d**,**f**) mesenteric lymph nodes (MLN) were determined at the indicated times after the first injection of smooth liposomes or of NPA-bearing liposomes. One experiment representative of three is shown. * *p* < 0.05; ** *p* < 0.01; SL, smooth liposomes; NPA-L, liposomes bearing promazine-induced NPAs.

We also evaluated the production of IFN-γ, IL-4 and IL-17 in NK1.1^+^, CD4^+^ T cells from C57BL/6 mice that produce IgG anti-NPA antibodies. IL-4-producing NK1.1^+^, CD4^+^ T cells from the spleen or mesenteric lymph nodes significantly increased, with *p* < 0.01 for spleens (Figure 5a) and *p* < 0.05 for mesenteric lymph nodes (Figure 5b), on days 20 and 30 after the first administration of NPA-bearing liposomes, compared to mice that received smooth liposomes. No significant differences were found in IFN-γ-producing NK1.1^+^, CD4^+^ T cells from the spleen (Figure 5c) or mesenteric lymph nodes (Figure 5d) after the administration of smooth or NPA-bearing liposomes. IL-17-producing spleen NK1.1^+^, CD4^+^ T cells (Figure 5e) were significantly increased (*p* < 0.05) on day 30 after the first administration of NPA-bearing liposomes. No significant differences were found in IL-17-producing NK1.1^+^, CD4^+^ T cells from mesenteric lymph nodes (Figure 5f) after the administration of the lipid antigens.

**Figure 6:**
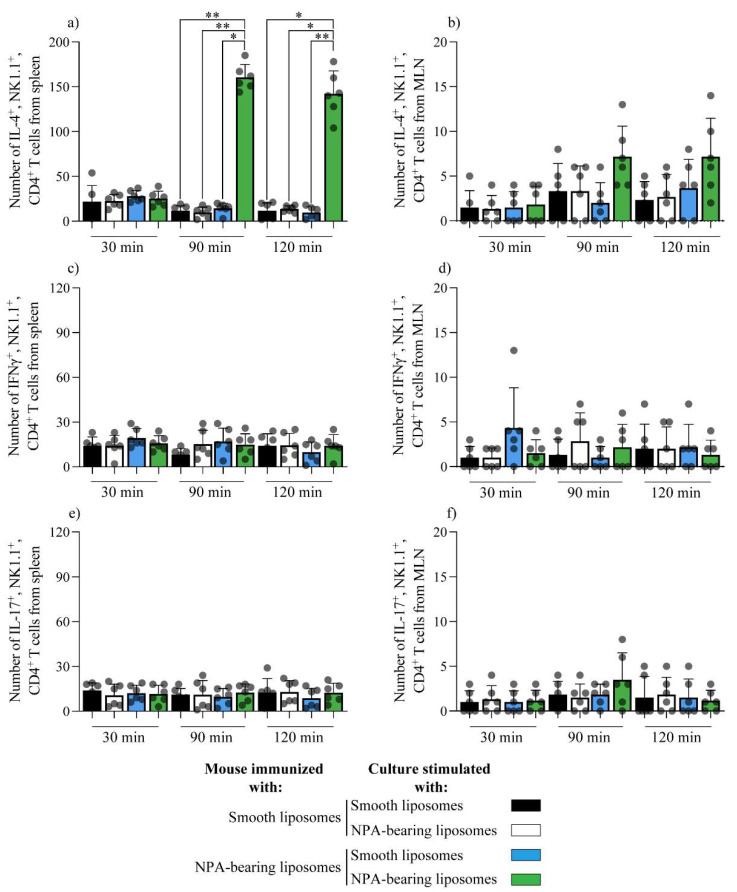
IL-4 production in response to NPAs by NK1.1^+^, CD4^+^ T cells from mice that produce IgG anti-NPA antibodies. Numbers of (**a**,**b**) IL-4-, (**c**,**d**) IFN-γ− or (**e**,**f**) IL-17-producing NK1.1^+^, CD4^+^ T cells from the (**a**,**c**,**e**) spleens or the (**b**,**d**,**f**) mesenteric lymph nodes of mice injected with smooth liposomes or with NPA-bearing liposomes, after re-stimulation of the cell suspensions with smooth liposomes or with NPA-bearing liposomes for 30, 90, or 120 min. One experiment representative of three is shown. The black dots represent the individual data results. * *p* < 0.05; ** *p* < 0.01; SL, smooth liposomes; NPA-L, liposomes bearing promazine-induced NPAs.

To determine if NK1.1^+^, CD4^+^ T cells could produce cytokines directly in response to NPAs, we evaluated the in vitro response of NK1.1^+^, CD4^+^ T cells from the spleens or mesenteric lymph nodes of mice that produce IgG anti-NPA antibodies, 20 days after the first administration of NPA-bearing liposomes. At 90 (*p* < 0.05) and 120 (*p* < 0.01) min after the re-stimulation with NPA-bearing liposomes, a statistically significant increase in NK1.1^+^, CD4^+^ T cells that produce IL-4 was detected in spleen cells from mice immunized with NPA-bearing liposomes, compared to the same cells re-stimulated with smooth liposomes, or compared with spleen cells from mice administered with smooth liposomes and re-stimulated with smooth liposomes or with NPA-bearing liposomes (Figure 6a).

This statistically significant increase in NK1.1^+^, CD4^+^ T cells that produce IL-4 was not detected at 30 min in spleen cells (Figure 6a), or at any time in the cells from mesenteric lymph nodes (Figure 6b). No significant differences were found in the production of IFN-γ (Figure 6c,d) or IL-17 (Figure 6e,f) by NK1.1^+^, CD4^+^ T cells from the spleen (Figure 6c,e) or mesenteric lymph nodes (Figure 6d,f) from mice administered with smooth liposomes or with NPA-bearing liposomes, when they were re-stimulated with smooth liposomes or with NPA-bearing liposomes.

### 3.3. NK1.1^+^, CD4^+^ T Cells from Mice That Produce IgG Anti-NPA Antibodies Over-Express Genes Related to the Immune Response

**Figure 7:**
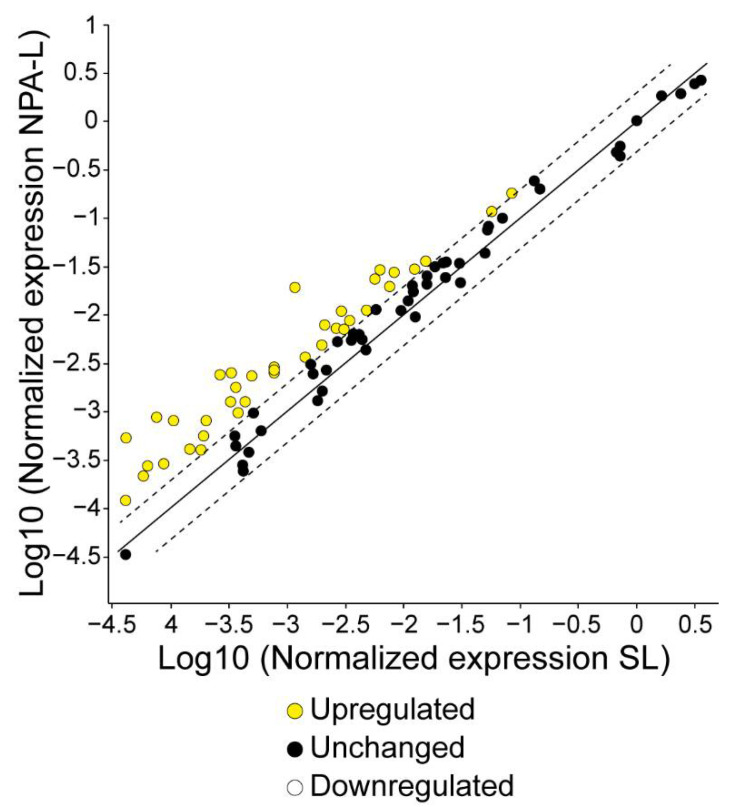
Gene expression profile of NK1.1^+^, CD4^+^ T cells from the spleens of mice that produce IgG anti-NPA antibodies. The expression levels of 84 genes related to the innate and adaptive immune responses were analyzed in NK1.1^+^, CD4^+^ T cells from the spleens of mice that received smooth liposomes (SL), and from the spleens of mice that received liposomes bearing promazine-induced NPAs (NPA-L). The scatter plot compares the normalized expression of each gene between the NPA-L and the SL groups by plotting them against one another, to visualize large gene expression changes. The central line indicates unchanged gene expression. The dotted lines indicate the selected fold regulation threshold.

To further confirm that NK1.1^+^, CD4^+^ T cells are activated in mice that produce IgG anti-NPA antibodies, we analyzed the gene expression of these cells in the spleens of mice, 20 days after the first administration of NPA-bearing liposomes. Forty of the eighty-four analyzed genes showed a difference in expression, compared with NK1.1^+^, CD4^+^ T cells from mice that received smooth liposomes. The 10 genes with a higher expression fold change were, in decreasing order, *Il1b*, *Ifnb1*, *Foxp3*, *Il10*, *Tlr9*, *Nlrp3*, *Cd8a*, *Ccr8*, *Cd86* and *Il6* (Table 1). No down-regulated genes were observed in this analysis (Figure 7).

These genes were selected by a fold regulation threshold of ≥2. The first ten genes (highlighted in blue) were used for a further analysis of the protein–protein interaction network.

**Figure 8:**
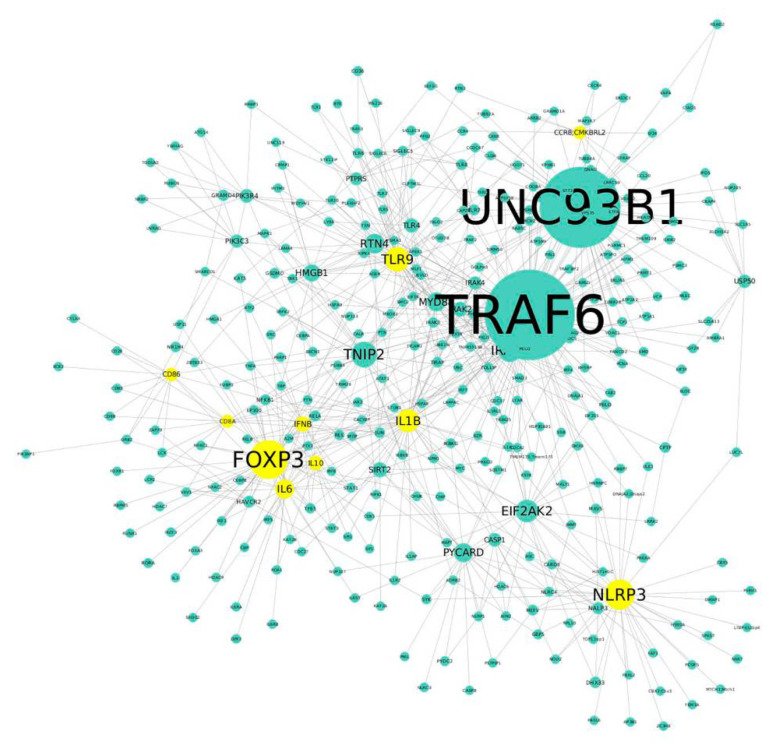
Protein–protein interaction network showcasing the relationship among the genes that are up-regulated in NK1.1^+^, CD4^+^ T cells from mice that produce IgG anti-NPA antibodies. The ten top up-regulated genes are highlighted in yellow. The size of the circles in the network is determined by the connectivity degree of each member of the network. *TRAF6* and *UNC93B1* are the most important nodes of this network.

An analysis of the top 10 up-regulated genes with the STRING and Cytoscape softwares revealed a network composed of these 10 genes and 1496 additional genes; the 10 genes that we identified were among the top 29 hub nodes with a high degree of interaction. We then selected only the main nodes, by selecting those with more than one interaction among members of the network, to analyze 300 nodes. The main nodes in this network were TRAF6 and UNC93B1 (Figure 8). *Traf6* was among the 84 genes that we analyzed in the array, but it had a fold change of 1.96 (the threshold chosen was 2), and *Unc93b1* was not included among these 84 genes. Both nodes also showed a high betweenness value, which makes them the most important nodes in the network, along with FOXP3, which was the best-ranked node and thus the most connected in the network from the 10 up-regulated genes revealed by the array (Table 2 and Figure 8).

## 4. Discussion

In a cell membrane matrix, the arrangement of phospholipids into bilayer is non-immunogenic. However, when the phospholipids re-arrange in stable NPAs, they become immunogenic and induce the production of IgG anti-NPA antibodies in mice. This re-arrangement can be generated in liposomes by drugs [17], such as chlorpromazine, promazine, procainamide or hydralazine, which produce as a side effect a lupus-like disease in humans [32]. The interaction between these drugs with anionic and conical shape phospholipids, like phosphatidate, generates a molecular re-arrangement that leads to the formation of an inverted micelle inserted into the lipid bilayer, which gives rise to the formation of a NPA. Consequently, the phospholipid polar heads in the outer region of the NPA spread, and new antigens are exposed and induce the production of IgG anti-NPA antibodies that trigger a lupus-like disease in mice [10,11]. These antibodies are also found in some patients with SLE [10].

The IgG anti-NPA antibodies are mainly generated by germinal center B cells [11]. However, the cell subset that helps these B cells to induce their activation and antibody production has not been yet identified. Several reports indicate that NKT cells are the T cell subset that participates in the response against lipid antigens [20,23]. After lipid antigen-specific activation, CD4^+^ NKT cells express co-stimulation molecules, such as CD40L, and produce Th1 (IFN-γ) or Th2 (IL-4) cytokines, so they provide the signals required for B cell activation and production of IgG antibodies against lipids, such as α-galactosylceramide [20,33]. Therefore, NKT cells could provide the necessary help for B cell maturation with the production of IgG anti-NPA antibodies in the mouse lupus-like model induced by NPAs, a lipid antigen in a special molecular association.

To study the potential role of NK1.1^+^, CD4^+^ T cells in this mouse model of lupus, we firstly induced the production of IgG anti-NPA antibodies in C57BL/6 mice using liposomes bearing promazine-induced NPAs. These are the hallmark antibodies of the BALB/c and NIH lupus models induced by NPAs [11]. We decided to use the C57BL/6 strain because of its high susceptibility to develop lupus; mice with the H-2^b^ haplotype, such as C57BL/6, have a higher risk of developing lupus nephritis, compared to mice with the H-2^d^ haplotype, such as BALB/c mice [34,35]. In addition to the production of IgG anti-NPA antibodies, C57BL/6 mice produced anti-cardiolipin and anti-histone antibodies, and also showed some of the first manifestations of the murine lupus-like disease [11,17,18], such as alopecia, deformation of limbs and facial lesions. These findings indicate that these mice are starting to develop the lupus-like disease in a similar way as has been described in BALB/c and NIH mice.

Furthermore, as NKT cells (CD3^+^, CD4^+^, NK1.1^+^) play an important role in the pathogenesis of autoimmune diseases like SLE [36], we analyzed NK1.1^+^, CD4^+^ T cells in the secondary lymphoid organs of C57BL/6 mice that produce IgG anti-NPA antibodies, and found a significant increase in the number of these cells in the spleen and mesenteric lymph nodes. These NK1.1^+^, CD4^+^ T cells mainly produced IL-4 in the mice with anti-NPA antibodies, and they also produced this cytokine after in vitro re-stimulation with NPAs. IL-4 is a cytokine that is involved in B cell maturation, survival, antibody production and class-switching [37,38]. Moreover, NK1.1^+^, CD4^+^ T cells that produce IL-17 were also detected in the spleens of mice that produce anti-NPA antibodies. IL-17 is a pro-inflammatory cytokine that has a strong influence in the development of adaptive immune responses, including autoimmunity, and it plays an important role in SLE development [39]. Finally, NK1.1^+^, CD4^+^ T cells did not produce IFN γ in mice with anti-NPA antibodies.

The significant increase in NK1.1^+^, CD4^+^ T cells suggests that these cells could participate in the activation of the B cells that produce IgG anti-NPA antibodies. The participation of CD4 in the activation of mice or human iNKT cells has been evaluated by Thedrez et al. [40], who observed a decrease in the production of IFN-γ and IL-4 when iNKT cells were activated with α-GalCer/CD1d in the presence of an anti-CD4 antibody. This suggests that CD4 binds to α-GalCer/CD1d on B cells and plays an important role in the effector functions of iNKT cells, such as the production of Th2- or Th1-type cytokines. In a similar way, NK1.1^+^, CD4^+^ T cells from mice that produce IgG anti-NPA antibodies could bind to NPA-CD1d on B cells and trigger the production of IL-4, the cytokine that is involved in antibody production and class-switching of B cells, as mentioned before [37,38]. Interestingly, in a previous study we showed that IL-4 KO BALB/c mice showed minor lupus-like disease manifestations, compared to wild-type BALB/c mice, with decreased production of IgG anti-NPA antibodies, absence of anti-cardiolipin, anti-histone and anti-coagulant antibodies, and no kidney or skin lesions. These findings indicate that IL-4 has a central role in the development of the murine lupus-like disease induced by stable NPAs [41], and they are a strong support to the findings of the present work referent to the production of IL-4 by NK1.1^+^, CD4^+^ T cells from C57BL/6 mice.

The expression of CD40L on NKT cells is associated with the lipid antigen activation of these cells, since blocking CD40L with a monoclonal antibody leads to reduced production of IgG, IgM and IgA antibodies, as well as IgG anti-dsDNA antibodies, in a co-culture of NKT cells and autologous B cells from lupus patients [23]. This implies that the production of antibodies requires the interaction of CD40 (on B cells) with CD40L (on NKT cells) [23]. In accordance with this information, we found a significant increase in the number of NK1.1^+^, CD4^+^, CD40L^+^ T cells in the spleens and mesenteric lymph nodes of mice that produce IgG anti-NPA antibodies. We can conclude that NK1.1^+^, CD4^+^ T cells in secondary lymphoid organs, as part of their adaptive functions, were activated in response to NPAs, so they expressed the CD40L molecule required to activate B cells, which, in turn, produced IgG anti-NPA antibodies.

To further characterize the NK1.1^+^, CD4^+^ T cells from mice that produce IgG anti-NPA antibodies, we evaluated their gene expression profiles. In these cells, forty genes were over-expressed, and the top ten genes with the highest expression, in decreasing order, were *Il1b*, *Ifnb1*, *Foxp3*, *Il10*, *Tlr9*, *Nlrp3*, *Cd8a*, *Ccr8*, *Cd86* and *Il6*. Foxp3 has hundreds of target genes in humans and mice, because it participates in many cellular processes, including differentiation and migration [42], but its most studied role is in regulatory T cells [43]. Engelmann et al. reports the expression of the *Foxp3* gene and its protein in CD4^+^, CD4^−^ CD8^−^ and CD8^+^ iNKT subsets from healthy donors, with the highest expression in CD4^+^ iNKT cells [44].

NLRP3 participates in the formation of the inflammasome, which activates the pro-inflammatory cytokines IL-1β and IL-18 [45]. The genes of these two cytokines were also over-expressed in the NK1.1^+^, CD4^+^ T cells from mice that produce IgG anti-NPA antibodies, and the IL-1β gene was the most over-expressed gene detected in these mice. The binding of lipopolysaccharide to TLR-4 can induce the transcription of *Nlrp3* [46]. In a previous study, we showed that NPA-bearing liposomes induce TLR-4 signaling in TLR-expressing HEK cells and also in bone marrow-derived mouse macrophages [18], so the binding of NPAs to TLR-4 on NK1.1^+^, CD4^+^ T cells could also increase the expression of NLRP3, which could contribute to the inflammation that precedes the adaptive immune response that produces the IgG anti-NPA antibodies.

The other seven top over-expressed genes in NK1.1^+^, CD4^+^ T cells (*Ifnb1*, *Il10*, *Tlr9*, *Cd8a*, *Ccr8*, *Cd86* and *Il6*) are probably involved in the activation of these cells through their TCR or TLR-4, or through cytokines such as IL-4, IL-6 and IL-13. It is well-known that the antigen-specific signal mediated by the TCR requires another antigen-independent signal provided by co-stimulation molecules for T cell activation [47]. The binding of the co-stimulation molecule CD40L on NKT cells with CD40 on B cells triggers the production of IL-4, IL-6, IL-13 and CD86, which are necessary to induce the response of B cells [27,47]. It should be noted that, in this work, CD40L was increased on NK1.1^+^, CD4^+^ T cells, and the *Il6* and *Cd86* genes were also over-expressed on these cells. These molecules could provide the signals required by B cells to produce IgG anti-NPA antibodies. TLR-4 signaling induces the expression of *Il1b*, *Il6*, *Il10* and *Ccr8* [48,49], suggesting a mechanism by which NPA-bearing liposomes could induce the expression of these genes on NK1.1^+^, CD4^+^ T cells. Over-expression of *Ccr8* has been previously reported on activated CD4^+^ NKT cells, and is considered a specific Th2 response gene [49,50].

Taking together all this information, we propose that, as part of their innate function, NK1.1^+^, CD4^+^ T cells recognize NPAs through their TLR-4, causing an increase in their expression of the *Il1b*, *Il6*, *Ccr8* and *Nlrp3* genes that participate in the development of the inflammatory response that activates and recruits cells of the adaptive immune response, and this could contribute to the production of IgG anti-NPA antibodies. We previously found an increase in the concentration of IL-6 and IL-10 in the serum of mice with the lupus-like disease induced by NPAs [18], which could indicate that NK1.1^+^, CD4^+^ T cells are involved in the production of these cytokines. In addition, an increase in the serum concentration of IFN-I (IFN-β) has also been described in this lupus-like mouse model [18], and this cytokine could induce the expression of *Ifnb1* and *Tlr9* [51,52,53], which were also found here to be over-expressed in the NK1.1^+^, CD4^+^ T cells from mice that produce IgG anti-NPA antibodies. A network analysis of the top 10 up-regulated genes in these NK1.1^+^, CD4^+^ T cells revealed that TRAF6 and UNC93B1 are the two main nodes of the network. TRAF6 is essential for the activation, survival and differentiation of CD4^+^ T cells. Currently, the function of TRAF6 in NKT cells is poorly understood [54,55]. However, it has been described that TRAF6 is necessary for the signaling of IL-1, CD40 and lipopolysaccharide [56]. UNC93B1 participates in the TLR-mediated response to nucleic acids, and it stabilizes TLRs during their transport to and from endosomes. UNC93B1 is required in TLR signaling to enable the production of IL-6, CD40, CD80 and CD86, and it controls the response of TLR7 and TLR9 [57,58]. UNC93B1 expression is up-regulated in SLE patients, and it is important for the optimal production of autoantibodies in lupus-prone mice (B6-Fas^lpr^ and BXSB) [59]. All these data underline the possible role of TRAF6 and UNC93B1, through their protein networks, on the production of IgG anti-NPA antibodies, a role that needs further study in the mice that produce these antibodies.

## 5. Conclusions

The increased numbers of NK1.1^+^, CD4^+^ T cells, their activation (CD40L^+^) and their production of IL-4 in the spleen and mesenteric lymph nodes of mice administered with NPAs, argue for their participation in the cooperation required for specific B cell activation and antibody class-switching in the production of IgG anti-NPA antibodies. Moreover, the NK1.1^+^, CD4^+^ T cells from the spleens of these mice over-expressed 40 genes associated with inflammation and with B cell stimulation, and these genes have TRAF6 and UNC39B1 as key nodes in their network. Altogether, the results of this work show that liposomes can be used to analyze the cells involved in the production of IgG antibodies against membrane phospholipids that are in a different molecular association to the membrane lipid matrix.

As future perspectives, we propose to analyze, in situ, the co-stimulation between the CD40L of NK1.1^+^, CD4^+^ T cells and the CD40 of NPA-specific germinal center B cells. We also propose to identify other cells that participate in lipid-antigen specific germinal centers (such as follicular dendritic cells and follicular T-lineage cells), the cytokines produced by these cells and their gene expression profiles. This knowledge would be useful for the rational design of vaccines against lipid antigens, and of vaccines that use liposomes as adjuvants.

## Figures and Tables

**Table 1:** Over-expressed genes of spleen NK1.1^+^, CD4^+^ T cells from mice that produce IgG anti-NPA antibodies.

Genes	Fold Regulation	Genes	Fold Regulation	Genes	Fold Regulation	Genes	Fold Regulation
*Il1b*	16.62	*Cxcl10*	4.16	*Il18*	3.34	*Tnf*	2.58
*Ifnb1*	13.04	*Mpo*	4.08	*Tlr2*	3.29	*Ifnar1*	2.56
*Foxp3*	11.82	*Cd40*	3.88	*Il2*	2.97	*Ly96*	2.47
*Il10*	9.08	*Il1a*	3.86	*Il23a*	2.97	*Cd4*	2.38
*Tlr9*	7.76	*Tlr4*	3.82	*Myd88*	2.94	*Icam1*	2.33
*Nlrp3*	7.71	*H2-Q10*	3.74	*Stat6*	2.81	*Casp1*	2.32
*Cd8a*	4.92	*Slc11a1*	3.72	*Tlr8*	2.80	*Mapk8*	2.30
*Ccr8*	4.72	*Tlr7*	3.72	*Irf7*	2.61	*Csf2*	2.24
*Cd86*	4.68	*Cd80*	3.51	*Nod2*	2.58	*Nfkbia*	2.15
*Il6*	4.38	*Tlr5*	3.40	*Mx1*	2.58	*Ifng*	2.04

**Table 2:** Principal proteins of the protein–protein interaction network from the ten top up-regulated genes of spleen NK1.1^+^, CD4^+^ T cells from mice that produce IgG anti-NPA antibodies.

Protein Name	Betweenness Value	Connectivity Degree	Protein Name	Betweenness Value	Connectivity Degree	Protein Name	Betweenness Value	Connectivity Degree
TRAF6	0.45664152	393	PYCARD	0.06525022	49	CASP1	0.01519920	22
UNC93B1	0.37356461	348	MYD88	0.03781771	48	USP50	0.01586547	21
FOXP3	0.13193952	142	IL6	0.03542776	47	PIK3C3	0.02003203	20
NLRP3	0.0972960	101	HMGB1	0.03793161	39	CD86	0.01148624	20
TNIP2	0.08172416	81	SIRT2	0.03014661	29	IRAK4	0.00940748	20
TLR9	0.14978064	74	IFNB	0.01508672	29	PTPRS	0.01635204	19
IRAK1	0.09756521	69	IRAK2	0.02226735	28	CCR8	0.01400334	19
EIF2AK2	0.06857739	66	TLR4	0.01940404	26	CD8A	0.01217994	18
IL1B	0.07191164	65	IL10	0.01699116	25	NALP3	0.00752642	15
RTN4	0.06375998	52	PIK3R4	0.02006215	23			

The connectivity degree and the betweenness value of each node is indicated for each protein. The proteins of the ten top up-regulated genes are highlighted in yellow.

## Data Availability

The data presented in this study are available on request from the corresponding authors.

## Abbreviations

MHC: Major histocompatibility complex, MLN: Mesenteric lymph nodes, NPA-L: Liposomes bearing promazine-induced NPAs, NPAs: Non-bilayer phospholipid arrangements, PA: L-α-phosphatidic acid, PC: L-α-phosphatidylcholine, PPI: Protein–protein interaction, SL: Smooth liposomes, SLE: Systemic Lupus Erythematosus, TCR: T cell receptor.
